# Assessment of Maropitant Citrate Effectiveness as an Intraoperative Analgesic Through Monitoring Parasympathetic Tone Activity in Female Dogs Undergoing Ovariohysterectomy

**DOI:** 10.3390/vetsci13050463

**Published:** 2026-05-10

**Authors:** Areli Ramírez-Castillo, Claudia Interlandi, Agatha Elisa Miranda Cortés, Navid Ziaei-Darounkolaei, Alejandro Casas-Alvarado, Alejandro Jiménez-Yedra, Ismael Hernández-Avalos

**Affiliations:** 1 Clinical Pharmacology and Veterinary Anesthesia, Biological Sciences Department, Facultad de Estudios Superiores Cuautitlán, Universidad Nacional Autónoma de Mexico (UNAM), Cuautitlán 54714, Mexico; 318245159@comunidad.unam.mx; 2 Department of Veterinary Sciences, University of Messina, 98168 Messina, Italy; cinterlandi@unime.it; 3 Pharmacology and Toxicology of Substances of Veterinary Interest, Universidad Nacional Autónoma de México, Cuautitlán 54714, Mexico; agatha.miranda@cuautitlan.unam.mx; 4 Department of Surgery and Radiology, Faculty of Veterinary Medicine, Bab.C., Islamic Azad University, Babol 47167-13311, Iran; ziaei@baboliau.ac.ir; 5 Department of Biological Sciences, Facultad de Estudios Superiores Cuautitlán, Universidad Nacional Autónoma de Mexico (UNAM), Cuautitlán 54714, Mexico; alejandro.casas@cuautitlan.unam.mx; 6 Department of Anesthesiology in Dogs and Cats, Specialty Veterinary Hospital, Facultad de Medicina Veterinaria y Zootecnia (FMVZ), Universidad Nacional Autónoma de México, Mexico City 04510, Mexico; ajyedra@unam.mx

**Keywords:** acute pain, nociception, analgesic adjuvant, dogs, analgesia

## Abstract

Maropitant has been proposed as an adjunct for pain relief in dogs undergoing surgeries like ovariohysterectomy (OVH), but its effectiveness has not yet been definitively proven. This study aimed to assess the intraoperative analgesic effect of intravenously administered maropitant citrate at a constant rate infusion through monitoring parasympathetic tone activity in female dogs undergoing OVH. Thirty healthy females of various breeds, with an average age of 3.8 ± 2.7 years, an average weight of 16.75 ± 10.68 kg, were randomly assigned to two treatment groups. The group receiving maropitant (G_Maro_, *n* = 15) was given a 1 mg kg^−1^ maropitant bolus intravenously (IV), followed by a continuous infusion of 100 mcg kg^−1^ min^−1^. The lidocaine group (G_Lido_, *n* = 15) received a 2 mg/kg lidocaine IV bolus, with subsequent infusion at 50 mcg kg^−1^ min^−1^. Cardiorespiratory variables and the PTA index were evaluated at 11 anesthetic time points. Overall, cardiovascular variables such as Heart Rate (HR) and systolic arterial pressure (SAP) significantly decreased during anesthesia induction in the G_Maro_ (*p* = *0.0001*; *p* = *0.01,* respectively) and in G_Lido_ (*p* = *0.01*). Differences between groups during induction were observed in HR (*p* = *0.03*), SAP (*p* = *0.04*), and mean arterial pressure (MAP) (*p* = *0.03*). MAP showed significant changes from baseline at the start of surgery and during clamping in both G_Maro_ (*p* = *0.03*) and G_Lido_ (*p* = *0.003*). Regarding ventilatory variables—pulse oximetry (SpO_2_), respiratory rate (RR), inspired oxygen fraction (FiO_2_), end-tidal CO_2_ (EtCO_2_)—no group differences were found, but RR (G_Maro_; *p* = *0.001*, G_Lido_; *p* = *0.0001*) and SpO_2_ (G_Maro_; *p* = *0.004*, G_Lido_; *p* = *0.04*) differed significantly from baseline due to the controlled clinical setting. During anesthesia maintenance, end-tidal isoflurane (ET_Iso_) increased significantly in the G_Lido_ (*p* = *0.009*), with no difference between groups (*p* = *0.94*). Finally, only the PTA energy variable showed a significant decrease in the G_Maro_ (*p* = *0.0006*), and a significant difference in this parameter was observed during right ovarian pedicle manipulation between groups (*p* = *0.02*). In conclusion, continuous intravenous infusion of maropitant citrate at 100 mcg kg^−1^ h^−1^ effectively reduced the sympathetic response related to nociception, similar to lidocaine, in healthy female dogs undergoing OVH.

## Simple Summary

The use of adjuvants as part of multimodal pain management during canine anesthesia has been recommended in recent years. This study evaluated the intraoperative analgesic efficacy of maropitant citrate administered as a constant rate infusion in female dogs undergoing ovariohysterectomy (OVH). Thirty female dogs with anesthetic risk ASA1 were evaluated; maropitant or lidocaine was administered via continuous infusion throughout the anesthetic procedure, and cardiorespiratory variables and the parasympathetic tone activity (PTA) index were assessed. Overall, no changes were observed in cardiorespiratory parameters, which remained within the normal ranges for a patient under general anesthesia; furthermore, the PTA index did not show variations associated with nociception. Therefore, the intravenous administration of maropitant citrate at a constant rate of 0.1 mg/kg/h successfully minimized the sympathetic response associated with nociception, similar to that observed with lidocaine, in healthy female dogs undergoing OVH.

## 1. Introduction

Nociception is an unconscious physiological response triggered by a harmful stimulus, engaging complex neural pathways that include peripheral receptors, the spinal cord, the thalamus, and the cerebral cortex [1,2,3]. The physiological response to nociception is a concern in anesthetic management in dogs because it can trigger neuroendocrine and metabolic responses, including increased catecholamine and cortisol levels. These responses can negatively affect the metabolism of carbohydrates, lipids, and proteins, and may lead to vasoconstriction [4,5]. This phenomenon is known as surgical stress, and because of the changes it induces, it could extend the patient’s recovery period [6,7], underscoring the importance of adequate analgesia in the perioperative period [8,9,10].

The effects of nociception during the perioperative period highlight the importance of managing this sign, not only to ensure animal welfare but also to enhance the quality of anesthetic recovery and postoperative care [11]. Opioids are the most frequently used drugs for pain management [5,12,13]. Currently, the use of these medications is restricted due to concerns about adverse effects and legal restrictions in some countries due to public health concerns [14].

Strategies pursued to reduce the use of opioids include multimodal analgesia, which involves using two or more drugs from different classes to achieve pain control through various neurobiological pathways [15]. One of these therapeutic options involves using adjuvant drugs—medications not originally intended for pain relief [16,17]. These drugs can modulate certain neurobiological pain pathways because of their mechanism of action. Research indicates that these adjuvants, like maropitant citrate, might be integrated into multimodal analgesia approaches [18,19].

Maropitant citrate is primarily approved as an antiemetic for dogs and cats, acting by antagonizing neurokinin 1 (NK-1) receptors [20]. Substance P, the endogenous ligand for these receptors, is a widely present neuropeptide in both the central and peripheral nervous systems. It is released at primary afferent nerve endings and plays a key role in the transmission of nociceptive stimuli and in mediating inflammatory processes [21,22]. Some studies in dogs indicate that maropitant provides postoperative pain relief when combined with other medications [23,24,25,26]. It has also been noted that using this medication together may reduce the requirements for anesthetic agents such as sevoflurane and desflurane [27,28], which would reaffirm its value in the comprehensive management of pain.

Although maropitant offers a clear benefit, there is limited evidence to confirm its effectiveness in managing perioperative nociception. This could potentially be established through innovative approaches, such as monitoring the parasympathetic tone activity (PTA) index, which has demonstrated utility in assessing the balance of the Autonomic Nervous System (ANS) [29]. This study aimed to assess the intraoperative analgesic effect of intravenously administered maropitant citrate at a constant rate infusion through monitoring parasympathetic tone activity in female dogs undergoing ovariohysterectomy (OVH). It was hypothesized that delivering maropitant citrate at a steady rate of 100 mcg kg^−1^ h^−1^ would offer effective pain control during surgery and reduce the sympathetic response to the surgical stimulus.

## 2. Materials and Methods

### 2.1. Animals

This study evaluated 30 female dogs of different breeds, with an average age of 3.8 ± 2.7 years, an average weight of 16.75 ± 10.68 kg, and a body condition score of 4/9 according to the body condition score system [30]. The sample size was estimated using G*power 3.1.9.7 software (Heinrich-Heine-Universität Düsseldorf, Düsseldorf, Germany). It was determined that the total sample size was 30 dogs, considering an alpha error probability (α) of 0.05, a confidence level of 95%, a power (probability of error 1 − α) of 0.90, and a correlation between repeated measures of 0.5.

All admitted animals underwent a complete general physical examination, complete blood count, serum biochemistry, and urinalysis to select healthy animals or those at ASA-1 anesthetic risk, according to the American Society of Anesthesiologists [31]. Animals presenting any condition that would cause acute pain, with an ASA-3 risk or higher, and those with any severe infectious disease were excluded. Anesthetic and surgical procedures were performed following written informed consent from the owners.

### 2.2. Experimental Design

The experimental study had a prospective, blind, and randomized design. A total of 30 female dogs were randomly assigned to two groups according to the treatment administered. G_Maro_ (*n* = 15) received maropitant bolus 1 mg kg^−1^ intravenously (IV) and continuous rate infusion (CRI) of 100 μg kg^−1^ min^−1^ [24]; and in G_Lido_ (*n* = 15) received lidocaine bolus at 2 mg kg^−1^ IV and CRI at 50 μg kg^−1^ min^−1^ [32].

The assessment of cardiorespiratory variables and PTA index was conducted at the following times: Baseline pre-surgery, considered 1 h before premedication (T_Basal_); sedation, considered 10 min after IV sedative administration (T_S_); Anesthetic Induction, considered 2 min after anesthesia induction (T_IN_); Anesthesia basal, considered 10 min start the anesthesia (T_Ba_); Pinching, clamping or placement of surgical field forceps (T_P_); Beginning of surgery; considered when surgical approach and first incision of the linea alba (T_BS_); Right Ovary ligation, when exteriorization and ligation of the right ovarian pedicle (T_RO_); Left Ovary ligation, when exteriorization and ligation of the left ovarian pedicle (T_LO_); uterine ligation, surgical transection of the uterine body (T_Ut_); Muscle suture, considered suturing of muscle planes and fasciae (T_Musc_); Suture, suturing of superficial planes and skin (T_Sut_); End of surgery (T_End_).

### 2.3. Anesthetic and Surgical Management

Study animals underwent a 4 h water fast and a 6 h food fast prior to anesthesia. Aseptic catheterization of the cephalic vein was performed using a 20 G × 31 mm or 22 G × 25 mm was inserted into the cephalic vein, depending on the animal’s weight, through which Hartmann’s solution was administered intravenously (IV) at a rate of 5 mL kg^−1^ h^−1^ (BeneFusion VP1 Vet, Mindray, Hamburg, Germany) during the surgical procedure [33].

After catheterization, Dexmedetomidine (Dexdomitor, Zoetis, Mexico) was administered intravenously at a dose of 2 µg kg^−1^. The dogs exhibited moderate sedation, which was assessed 10 min after sedative administration using the sedation scale proposed by Grint et al. [34]. Anesthetic induction was achieved with Propofol (Xendta, Troikaa Pharmaceuticals, India) at 2–3 mg kg^−1^ IV [35]. Orotracheal intubation was performed upon observation of ventromedial deviation of the eyeball and decreased mandibular tone. Subsequently, patients were connected to a rebreathing anesthetic circuit for administration of 100% oxygen at a flow rate of 45 mL kg^−1^ min^−1^. Anesthesia maintenance was achieved through the vaporization of isoflurane (Sofloran, Pisa, Mexico) with the dial set to 1.7%, adjusting this concentration according to the required anesthetic depth to maintain a mean arterial pressure (MAP) of 60–90 mmHg. The vaporizer was adjusted to achieve an end-expiratory isoflurane fraction (ET_Iso_) between 1.1 and 1.3% prior to surgery and was then adjusted to maintain anesthesia (no palpebral reflex, ventromedial ocular globe, and no mandibular tone). Throughout the anesthesia–surgical procedure, all animals were mechanically ventilated in pressure-controlled mode with an airway pressure (Paw) of 12–15 cmH_2_O during inspiration, an inspiratory-to-expiratory ratio (I/E) of 1:2, and an rise time of 0.6 s, using a mechanical ventilator integrated into the anesthesia station (Wato-EX20 vet, Mindray, Germany), with the settings adjusted to maintain an end-tidal carbon dioxide (ETCO_2_) level of 35–45 mmHg.

### 2.4. Intraoperative Monitoring

The evaluated parameters included heart rate (HR), recorded via electrocardiographic tracing in lead II; non-invasive blood pressure (NIBP), measured by oscillometry, from which systolic arterial pressure (SAP), diastolic arterial pressure (DAP), and mean arterial pressure (MAP) were obtained; pulse oximetry (SpO_2_), assessed by infrared spectrophotometry; respiratory rate (RR), measured by spirometry; and end-tidal carbon dioxide (ETCO_2_), monitored by capnography, and fraction of inspired oxygen (FiO_2_). Esophageal temperature (°C) was also recorded, and end-tidal isoflurane concentration (ET_Iso_) was measured using the same monitoring system (ePM12VETc/AA, Mindray, Hamburg, Germany). Finally, the perfusion index (Pi) and plethysmographic variability index (PVi) were obtained from co-oximetry (Radical 7 Rainbow SET, Masimo, Irvine, CA, USA).

Cardiovascular parameters (HR and NIBP), and esophageal temperature (°C) were obtained at times T_Basal_, T_S_, T_IN_, T_Ba_, T_P_, T_BS_, T_RO_, T_LO_, T_Ut_, T_Musc_, T_Sut_, and T_end_.

The RR, SpO_2_, Pi, and PVi were obtained at times T_Basal_, T_S_, T_IN_, and T_Surg_ (Intraoperative monitoring from the beginning to the end of surgery). FiO_2_, ETCO_2_, and ET_Iso_ were obtained at times T_IN_ and T_Surg_.

### 2.5. Parasympathetic Tone Activity (PTA) Index

Nociception was assessed using the Physio Doloris PTA monitor (MDoloris Medical Systems, Loos, France). This non-invasive device collects the patient’s lead II of the electrocardiographic tracing. The PTA index is calculated from the respiratory cycle’s influence on the RR interval of the electrocardiogram, enabling both qualitative and quantitative analysis of heart rate variability modulated by the autonomic nervous system [29,36]. Immediate PTA (PTAi) values were obtained from a continuous recording during surgery. This PTAi is recognized as the surgeon’s index and is calculated from high-frequency (0.15–0.5 Hz) or low-frequency (0.04–0.15 Hz) waves displayed in areas under the curve called A1, A2, A3, and A4, which form a continuous recording within a 64 s moving window. When the R-R series are normalized, they show an amplitude of 0–0.2 normalized units, fluctuating between −0.1 and 0.1 nu. This register indicates that a window of 16–64 s is used to calculate PTAi. On the other hand, the values of the mediate PTA index (PTAm) are recognized as the anesthesiologist’s index, which are collected during 120 s and 240 s. The clinical interpretation of the values for dogs is made according to the following score: 50–70 reflects adequate intraoperative analgesia to inhibit hemodynamic response, indicating that the patient is in a comfortable state; a score of 40–49 denotes mild to moderate nociception with possible hemodynamic reactivity secondary to insufficient analgesia; while a score of 0–39 indicates severe nociception [12,37,38].

The values of PTAi, PTAm, and energy measured in Hz were recorded at the same times as the cardiorespiratory parameters.

### 2.6. Rescue Analgesic-Antinociceptive Medication

If dogs had a PTAm value below 50 points, they also experienced a 20% increase in HR and MAP from baseline. A rescue bolus of butorphanol at 0.2 mg kg^−1^ was administered for analgesia (Butonil, WildLife Pharmaceuticals).

### 2.7. Statistical Analysis

To perform the statistical comparison of the variables instrumentation time, anesthesia time, surgery duration, and anesthetic recovery time, Student’s *t*-test was used.

Cardiorespiratory parameters and PTA variables were expressed for each study group (G_Maro_ and G_Lido_) as mean ± standard error across the eleven times of study. Data normality for all evaluated variables was assessed using the Kolmogorov–Smirnov test. All study variables were analyzed using a generalized linear mixed model (GLMM). The model used was as follows:

Yijk = μ + Ti + Fj + (TF)ij + Pk(Ti) + Ɛijk

where

Yijk = observed value of the variable in patient k, under treatment i, at surgical phase j;

μ = overall mean;

Ti = fixed effect of treatment (i = G_Maro_ and G_Lido_);

Fj = fixed effect of surgical phase (j = times T_Basal_, T_S_, T_IN_, T_Ba_, T_P_, T_BS_, T_RO_, T_LO_, T_Ut_, T_Musc_, T_Sut_, T_End_);

(TF)ij = interaction between treatment and surgical phase;

Pk(Ti) = random effect of each patient k nested within treatment i;

Ɛijk = random or residual error.

To assess differences between means, Tukey’s post hoc test was employed. Statistical significance was set at *p* < 0.05 in all comparisons. PRISM version 11.0.0 (Boston, MA, USA) was used for the statistical analysis.

### 2.8. Ethical Statement

All procedures involving animal management throughout the study complied with the requirements established by the Mexican Official Standard NOM-062-ZOO-1999, which outlines the technical specifications for the production, care, and use of laboratory animals. The project received approval from the Internal Committee for the Care and Use of Laboratory Animals (CICUAE, by the Spanish acronym) of the National Autonomous University of Mexico (registration number C25_21). In addition, this study was conducted in accordance with the ARRIVE guidelines and international directives for the ethical use of animals in experimentation [39,40]. No phase of the study, including the surgical procedure or data collection, resulted in animal injury, mutilation, or excessive handling.

## 3. Results

Table 1 presents the mean ± standard deviation (SD) for instrumentation time, anesthesia time, surgical duration, and anesthetic recovery time. No statistically significant difference was observed in any of these variables (*p > 0.05*).

**Figure 1:**
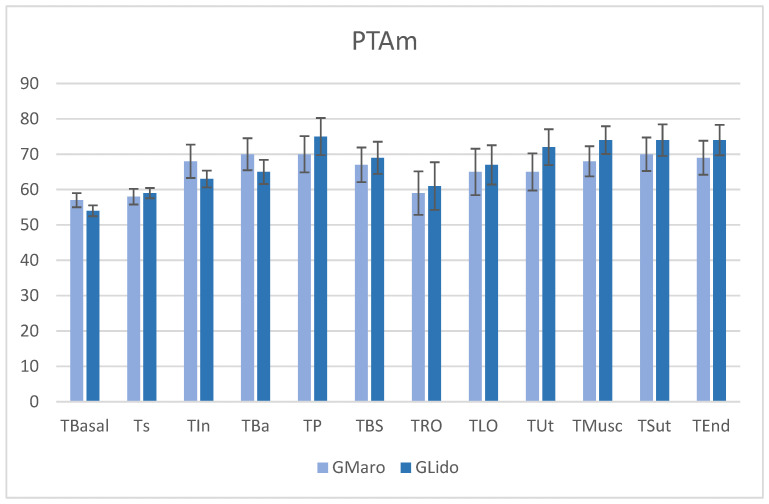
Mediate parasympathetic tone activity (PTAm) index assessed in G_Maro_ and G_Lido_ during different surgical-nociceptive times in female dogs undergoing ovariohysterectomy. No significant differences compared to T_Basal_, either between evaluation times (*p* = *0.99*) or between treatments (*p* = *0.99*). T_Basal_—baseline pre-surgery; T_S_—sedation; T_In_—anesthetic induction; T_Ba_—anesthesia basal; T_P_—pinching, clamping or placement of surgical field forceps; T_BS_—beginning of surgery; T_RO_—right ovary ligation; T_LO_—left ovary ligation; T_Ut_—uterine ligation and surgical transection; T_Musc_—muscle suturing; T_Sut_—suturing of superficial planes and skin; T_End_—end of surgery.

Figure 1 shows no significant differences between the time points and T_Basal_ (*p* = *0.99*), indicating that PTA remained stable throughout the surgical procedure. Both analgesic methods, G_Maro_ and G_Lido_, showed similar effectiveness in maintaining autonomic balance and preventing fluctuations due to nociception or surgical stress. During various surgical phases, there was a noticeable trend of increased PTAm, reflecting an appropriate balance between pain signals and analgesia. In potentially more painful situations, PTAm did not decrease, indicating an effective antinociceptive response. Although there was some data variability reflected in the error bars, this was not enough to achieve statistical significance (*p* = *0.99*). Consequently, both analgesic approaches successfully maintained autonomic stability during OVH, with no notable differences in PTA across surgical times or treatments, suggesting effective control of intraoperative nociception. The stable PTAm values imply that the animals remained in a balanced state between sympathetic and vagal activity.

**Figure 2:**
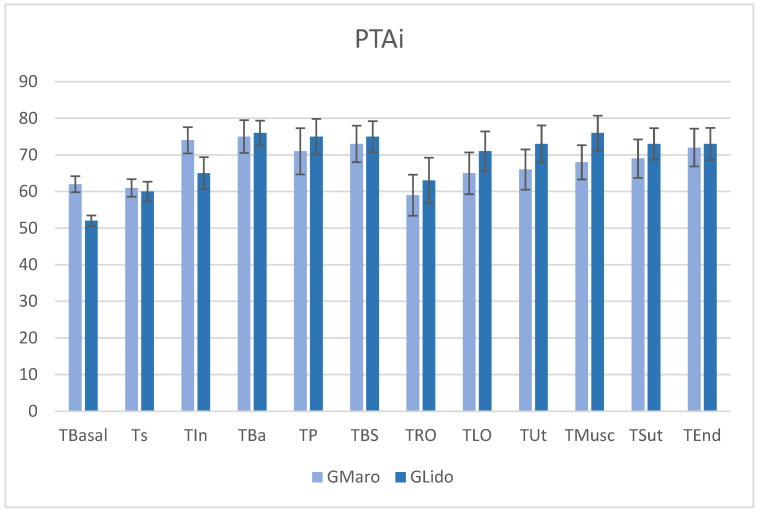
Immediate parasympathetic tone activity (PTAi) index assessed in G_Maro_ and G_Lido_ during different surgical-nociceptive times in female dogs undergoing ovariohysterectomy. No significant differences compared to T_Basal_, either between evaluation times (*p* = *0.76*) or between treatments (*p* = *0.99*). T_Basal_—baseline pre-surgery; T_S_—sedation; T_In_—anesthetic induction; T_Ba_—anesthesia basal; T_P_—pinching, clamping or placement of surgical field forceps; T_BS_—beginning of surgery; T_RO_—right ovary ligation; T_LO_—left ovary ligation; T_Ut_—uterine ligation and surgical transection; T_Musc_—muscle suturing; T_Sut_—suturing of superficial planes and skin; T_End_—end of surgery.

Figure 2 presents the PTAi values obtained during the same assessment points as the PTAm. Throughout these periods, the PTAi values (representing the anesthesiologist’s index) remained within the comfort zone (score 50–80), indicating hemodynamic and autonomic stability throughout the surgery. It is also evident that the treatment groups exhibit similar profiles at all assessment times. Statistical analysis shows no nociceptive effect between surgical times (*p* = *0.76*) or between the experimental groups (*p* = *0.99*). The PTAi results confirm that, during OVH in female dogs, nociception was adequately managed. Additionally, there are no differences between G_Maro_ and G_Lido_, suggesting both protocols offer comparable autonomic stability and clinical efficacy during surgery.

Based on hemodynamic monitoring during intraoperative nociception, no patient required analgesic rescue, as their cardiorespiratory parameters remained within normal ranges for anesthetized patients.

Table 2 presents the values of the cardiovascular parameters. HR decreased from baseline over time after general anesthesia in the G_Maro_ (*p* = *0.0001*) and G_Lido_ (*p* = *0.006*). In this variable, between treatments, the HR was that G_Maro_ was higher than G_Lido_ in the T_In_ (*p* = *0.03*) and T_Ba_ (*p* = *0.05*). Body temperature showed a statistically significant reduction in assessment times during surgery in G_Maro_ (*p* = *0.006*) and G_Lido_ (*p* = *0.04*).

SAP significantly decreased during anesthesia induction in the G_Maro_ (*p* = *0.0001*; *p* = *0.01,* respectively) and in G_Lido_ (*p* = *0.01*). Differences between groups during induction were observed in SAP (*p* = *0.04*) and mean arterial pressure (MAP) (*p* = *0.03*). MAP showed significant changes from baseline at the start of surgery and during clamping in both G_Maro_ (*p* = *0.03*) and G_Lido_ (*p* = *0.003*). On the other hand, energy showed a significant reduction in all anesthetic times compared to baseline in G_Maro_ (*p* = *0.0006*). Between treatments, G_Lido_ had higher energy than G_Maro_ in the T_RO_ (*p* = *0.02*).

Table 3 presents the ventilatory variables. FiO_2_, ETCO_2_, and Pi showed no significant differences from T_Basal_ (*p > 0.44*) or between treatments (*p* = *0.99*). However, RR (*p* = *0.001*) and SpO_2_ (*p* = *0.004*) differed significantly from T_Basal_ in both groups. In this case, PVi was significantly reduced during T_Surg_ in the G_Maro_ (*p* = *0.0003*). The ET_Iso_ was statistically different in G_Lido_, with a higher anesthetic requirement during surgery (*p* = *0.009*). However, there were no statistically significant differences between treatment groups in either T_IN_ (*p* = *0.24*) or T_Surg_ (*p* = *0.94*).

## 4. Discussion

The results of this study showed that G_Maro_ and G_Lido_ behaved similarly in PTAm, PTAi, cardiovascular variables, and ventilatory parameters. This suggests that maropitant citrate has analgesic properties similar to those of lidocaine, as indicated in various studies [25,26,41,42]. In this sense, lidocaine proved effective at the continuous infusion dose used, demonstrating its expected antinociceptive effect. The mechanism by which lidocaine induces analgesia is based on the blockade of Na+ ion channels, which causes a non-specific blockade of sensory and motor fibers [43,44]. However, maropitant blocks NK1 receptors for substance P; these receptors are present in the dorsal horn of the spinal cord, which modulates the transmission of noxious and peripheral sensory fibers involved in the transmission of impulses caused by noxious stimulation [27,45].

Todd et al. [46] reported that, in rats, neurons in the dorsal horn of Rexed laminae III and IV express NK-1 receptors containing substance P, and these same neurons project to regions of the brainstem and thalamus. Gautam et al. [47] suggest that these receptors are also found at the peripheral level and not only at the central level, so their interaction with both contributes to the modulation of pain, hyperalgesia, and the appearance of allodynia. This would reinforce the idea that blocking a pain sensation modulator can significantly contribute to analgesia.

The published evidence on the analgesic efficacy of maropitant citrate presents contrasting reports. For example, Silva et al. [41] observed that the use of maropitant at CRI of 50, 75, 100, and 200 mcg kg^−1^ h^−1^ was not effective in suppressing the autonomic responses to somatic and visceral nociceptive stimulation during OVH in dogs, so they concluded that this drug did not demonstrate any apparent effect on postoperative pain assessment. A possible explanation for these differences could be related to the blockade of the facilitation mechanism of NK-1 receptors in response to noxious stimuli. According to Khasabov et al. [48], the superficial neurons of the dorsal horn that express NK-1 receptors provide the excitatory impulse that activates facilitating mechanisms; that is to say, the excitation of neurons that express NK-1 could lead to a state in which activating neurons dominate activity in the rostral ventromedial medulla (RVM) and, therefore, produce facilitation of nociceptive transmission. However, an additional explanation for this difference in the effectiveness of maropitant citrate may be differences in NK-1 receptor density in dogs, which could result in differences in antinociceptive efficacy [49]. On the contrary, in rodents it has been shown that NK-1 receptors are abundant in the dorsal horn of the spinal cord, which could be an advantage when using this drug in this species [50,51], although there are similarities to dogs, since the distribution of receptors at the visceral level has also been observed [52,53].

In the present study, the lack of significant differences in PTAi and PTAm values in G_Maro_ and G_Lido_ may be due to these drugs being used perioperatively under a preventive analgesia strategy, given the IV administration of a bolus followed by a continuous infusion, which could have prevented autonomic changes resulting from sympathetic stimulation caused by surgical nociception [53,54]. Physiologically, substance P activates the pro-inflammatory master transcription factor nuclear factor kappa B (NF-κB) in NCM460 cells through mechanisms involving Rho and PKCδ kinases, leading to the production of IL-6, IL-8, and tumor necrosis factor α (TNF-α). Substance P also induces cyclooxygenase-2 expression and prostaglandin (PG) E2 production in these cells via the PKCθ and JAK3/STAT3/5 pathways. For this reason, perioperative administration of maropitant citrate helps reduce central sensitization that occurs after surgical procedures by selectively blocking NK-1 receptors [27,55]. These mechanisms have enabled the explanation of reduced analgesic requirements and the prevention of hyperalgesia or allodynia in some surgical models [45,56,57].

One of the parameters evaluated in the PTA monitor was energy level, which was higher in the G_Maro_. This parameter helps assess the balance between the sympathetic and parasympathetic components of the autonomic nervous system (ANS) [58]. Energy is determined in Hz and is obtained from the analysis of the frequency-domain components of heart rate variability (HRV), where the ratio of low-frequency to high-frequency components helps predict associated changes in hemodynamic stability [59,60]. Therefore, this parameter helps to understand the observed effect in this study, where maropitant citrate had a lower HRV, which suggests that blocking NK-1 receptors may help reduce central sensitization and, consequently, nociceptive changes.

On the other hand, lidocaine acts by blocking Na+ channels, which reduces the sympathetic vasomotor response by inhibiting sympathetic fibers that transmit nociceptive impulses [15,61,62]. Since vasomotor regulation is inhibited, the use of lidocaine is often associated with vasodilation phenomena, which could possibly be detected through a PTA monitor, not as a nociception phenomenon but as a hypotension response. According to Mansour et al. [29], dynamic variation in the PTA can predict hemodynamic response, even in human medicine, as the relationship between low- and high-frequency components can predict intraoperative hypotension [59]. In this way, the response observed in the dynamic variability of the PTA could not necessarily be interpreted as worse analgesic performance by lidocaine, since the rest of the physiological parameters remained within normal ranges without alterations associated with nociception.

The PVi variable is a non-invasive, preload-dependent measurement obtained from the pulse oximetry plethysmographic waveform. Therefore, the PVi value helps predict the vasomotor response [63]. In the present study, a statistically significant difference was observed in G_Maro_ but not in G_Lido_. This effect could again be attributed to the distribution of NK receptors, as they are present in the nervous system, genitourinary system, immune system, gastrointestinal tract, and cardiovascular system [64,65,66,67,68,69,70]. In this last case, Thomson et al. [71] determined the distribution of NK receptors and their relationship in cardiac regulation in dogs. In their study, they observed that NK-1 and NK-2 receptor agonists decreased left ventricular pressure, whereas an NK-3 receptor agonist increased neuronal activity in the right atrium. Therefore, blocking these receptors could prevent sympathetic stimulation, but it would also reduce vasomotor responses.

Chi et al. [72] reported that intravenous administration of maropitant caused significant decreases in SAP, DAP, and MAP at 16 min post-injection. The antagonist effect on substance P may reduce nitric oxide release from the vascular endothelium, thereby preventing relaxation of vascular smooth muscle [73]. This could possibly explain the decrease in peripheral flow observed in the PVi measurement in the G_Maro_ compared to the G_Lido_.

The requirement for isoflurane (ET_Iso_) showed no statistically significant differences between the groups during surgery (*p* = 0.94). However, in cats, maropitant citrate has been reported to reduce the minimum alveolar concentration (MAC) of sevoflurane by at least 15% [74]. In this study, both maropitant and lidocaine effectively prevented peripheral and central sensitization processes. Additionally, the anesthetic requirements for isoflurane stayed below the established MAC in dogs. Lidocaine’s effectiveness is attributed to its ability to inhibit the transduction of nociceptive signals [75], whereas maropitant’s is due to its modulation of these signals in the dorsal horn. This mechanism, independent of each drug, possibly prevents central nervous system activation and, consequently, keeps the hemodynamic response of the dogs in the study stable during OVH [1,76]. Continuous infusion (CRI) of analgesics reduces the end-tidal fraction of isoflurane and, in addition, stabilizes hemodynamic parameters, decreases the intraoperative response to noxious stimuli, and lessens postoperative pain intensity. Therefore, continuous infusion analgesia can be an effective strategy to ensure adequate pain relief, improve anesthesia, and minimize side effects [77].

The lack of differences in anesthetic requirements might be a limitation of this study. Specifically, not including a negative control group (placebo) to measure the percentage reduction in anesthetic is a shortcoming. Additionally, not having a positive control where multimodal analgesic treatment was used is another limitation. In other models, intraoperative analgesic monotherapy has been shown to be less effective in managing adrenocortical and glycemic responses (related to anesthetic-surgical stress) and postoperative pain, which can directly impact the quality of recovery [5]. This fact could potentially open up the possibility of proposing a study framework that compares treatments known to lack sparing effects relative to general anesthetics. Another limitation of this study is the patient selection, as results in healthy individuals may not be directly applicable to animals with pathological conditions, where treatment efficacy could differ. The absence of a pharmacokinetic profile, including parameters like Cmax, Tmax, and AUC, also limits the understanding of maropitant citrate’s analgesic adjuvant effects. Future studies could explore its analgesic impact in more invasive procedures, such as orthopedic or traumatic surgeries. Lastly, the lack of more sensitive and specific methods for monitoring hemodynamic parameters, like invasive blood pressure (IBP), is another potential limitation.

## 5. Conclusions

The intravenous administration of maropitant citrate at a constant rate of 100 mcg kg^−1^ h^−1^ successfully minimized the sympathetic response associated with nociception, resulting in a response similar to that of female dogs receiving lidocaine at a dose of 50 mcg kg^−1^ min^−1^ undergoing the surgical model of OVH.

## Figures and Tables

**Table 1:** Evaluation of instrumentation time, anesthesia time, surgical duration, and anesthetic recovery time in G_Maro_ and G_Lido_. Times are reported in minutes (mean ± SD).

	G_Maro_	G_Lido_
Instrumentation time	66.33 ± 10.66 a	66.53 ± 18.55 a
Anesthesia time	40.53 ± 9.90 a	41.06 ± 8.03 a
Surgical duration	27.93 ± 5.00 a	28.80 ± 3.98 a
Anesthetic recovery time	10.13 ± 2.47 a	9.20 ± 3.34 a

Letter “a” indicates that there was no statistically significant difference between study groups.

**Table 2:** Cardiovascular parameters (Mean ± SD) assessed in G_Maro_ and G_Lido_ during different surgical-nociceptive times (T) in female dogs undergoing ovariohysterectomy.

Variable	Group	T_Basal_	T_s_	T_In_	T_Ba_	T_P_	T_BS_	T_RO_	T_LO_	T_Ut_	T_Musc_	T_Sut_	T_End_	*p* Value Intragroup
**HR**	G_Maro_ n = 15	136 ^a,1^ ± 6.98	83 ^b,1^ ± 7.93	126 ^a^^,1^ ± 7.52	118 ^a^^,b,1^ ± 5.52	108 ^a^^,b1^ ± 3.88	113 ^a^^,b,1^ ± 4.42	100 ^b,1^ ± 4.27	98 ^b,1^ ± 3.19	100 ^b,1^ ± 3.50	97 ^b,1^ ± 3.89	97 ^b,1^ ± 4.10	95 ^b,1^ ± 5.89	***p*** ** = ** ***0.0001***
	G_Lido_ n = 15	135 ^a,1^ ± 6.34	83 ^b,1^ ± 6.78	73 ^b,2^ ± 5.77	79 ^b,2^ ± 5.53	92 ^b,1^ ± 3.90	88 ^b,1^ ± 4.84	93 ^b,1^ ± 4.96	92 ^b,1^ ± 4.63	91 ^b,1^ ± 3.89	89 ^b,1^ ± 3.97	89 ^b,1^ ± 3.22	90 ^b,1^ ± 3.13	***p *** **= ** ***0.006***
	*p* value groups	*p* = 0.99	*p* = 0.99	***p *** **= ** ***0.03***	*p* = 0.05	*p* = 0.66	*p* = 0.29	*p* = 0.99	*p* = 0.99	*p* = 0.98	*p* = 0.97	*p* = 0.98	*p* = 0.99	
**T °C**	G_Maro_ n = 15	38.81 ^a^^,1^ ± 0.13	38.76 ^a^^,1^ ± 0.17	38.26 ^a^^,b,1^ ± 0.20	38.15 ^a^^,b,1^ ± 0.21	37.83 ^b,c,d,e,1^ ± 0.24	37.87 ^a^^,b,c,1^ ± 0.24	37.74 ^c,d,e,1^ ± 0.24	37.67 ^d,e,1^ ± 0.25	37.63 ^d,e,1^ ± 0.26	37.57 ^d,e,1^ ± 0.24	37.49 ^e,1^ ± 0.26	37.49 ^e,1^ ± 0.26	***p *** **= ** ***0.006***
	G_Lido_ n = 15	38.81 ^a^^,1^ ± 0.15	38.57 ^a^^,1^ ± 0.18	38.17 ^a^^,1^ ± 0.20	37.96 ^a^^,b,1^ ± 0.23	37.22 ^d,1^ ± 0.50	37.87 ^a^^,b,c,1^ ± 0.24	37.13 ^d,1^ ± 0.50	37.50 ^b,c,1^ ± 0.31	37.41 ^b,c,1^ ± 0.31	37.41 ^b,c,1^ ± 0.31	37.27 ^d,1^ ± 0.32	37.31 ^d,1^ ± 0.33	***p *** **= ** ***0.04***
	*p* value groups	*p* = 0.99	*p* = 0.99	*p* = 0.99	*p* = 0.99	*p* = 0.99	*p* = 0.99	*p* = 0.99	*p* = 0.99	*p* = 0.99	*p* = 0.99	*p* = 0.99	*p* = 0.99	
**SAP** **(mmHg)**	G_Maro_ n = 15	139 ^a^^,1^ ± 9.44	154 ^a^^,1^ ± 10.63	140 ^a^^,1^ ± 8.98	133 ^a^^,1^ ± 8.40	110 ^a,1^ ± 6.28	119 ^a,1^ ± 7.39	140 ^a,1^ ± 6.29	136 ^a,1^ ± 4.64	133 ^a^^,1^ ± 5.05	118 ^a^^,1^ ± 4.83	119 ^a^^,1^ ± 4.67	120 ^a^^,1^ ± 3.82	*p* = 0.91
	G_Lido_ n = 15	140 ^a^^,1^ ± 7.32	129 ^a,b,1^ ± 7.98	97 ^b,c,2^ ± 5.11	114 ^a,b,1^ ± 10.90	105 ^a,b,1^ ± 6.80	107 ^a,b,1^ ± 7.51	131 ^a,1^ ± 8.79	132 ^a^^,1^ ± 9.65	129 ^a^^,b,1^ ± 9.06	114 ^a^^,b,1^ ± 9.71	116 ^a^^,b,1^ ± 8.90	112 ^a^^,b,1^ ± 8.88	***p *** **= ** ***0.01***
	*p* value groups	*p* = 0.99	*p* = 0.80	***p *** **= ** ***0.04***	*p* = 0.88	*p* = 0.99	*p* = 0.98	*p* = 0.99	*p* = 0.99	*p* = 0.99	*p* = 0.99	*p* = 0.99	*p* = 0.99	
**MAP** **(mmHg)**	G_Maro_ n = 15	109 ^a,b,1^ ± 6.35	125 ^a,1^ ± 8.16	113 ^a,1^ ± 7.35	104 ^a,b,1^ ± 7.63	83 ^b,c,1^ ± 5.34	91 ^a^^,1^ ± 7.12	111 ^a^^,b,1^ ± 4.70	113 ^a^^,1^ ± 4.84	102 ^a^^,b,1^ ± 4.19	90 ^a^^,b,1^ ± 3.55	89 ^a^^,b,1^ ± 3.27	91 ^a^^,b,1^ ± 3.24	***p *** **= ** ***0.03***
	G_Lido_ n = 15	109 ^a^^,1^ ± 7.52	100 ^a,b,1^ ± 7.52	73 ^b,2^ ± 5.65	87 ^a,b,1^ ± 10.16	77 ^a^^,b,1^ ± 5.96	81 ^a^^,b,1^ ± 7.25	102 ^a^^,1^ ± 8.16	107 ^a^^,1^ ± 8.90	102 ^a^^,1^ ± 8.24	88 ^a^^,b,1^ ± 8.83	91 ^a^^,b,1^ ± 8.17	89 ^a^^,b,1^ ± 7.68	***p *** **= ** ***0.003***
	*p* value groups	*p* = 0.99	*p* = 0.99	***p *** **= ** ***0.03***	*p* = 0.99	*p* = 0.99	*p* = 0.99	*p* = 0.99	*p* = 0.99	*p* = 0.99	*p* = 0.99	*p* = 0.99	*p* = 0.99	
**DAP** **(mmHg)**	G_Maro_ n = 15	98 ^a^^,1^ ± 7.43	111 ^a^^,1^ ± 6.99	98 ^a^^,1^ ± 7.03	88 ^a^^,b,1^ ± 7.27	71 ^b,1^ ± 6.74	75 ^b,1^ ± 6.99	95 ^a^^,1^ ± 6.21	98 ^a^^,1^ ± 4.68	89 ^a^^,1^ ± 4.11	76 ^b,1^ ± 3.48	74 ^b,1^ ± 3.09	77 ^b,1^ ± 3.15	***p *** **= ** ***0.01***
	G_Lido_ n = 15	91 ^a^^,1^ ± 5.76	87 ^a,1^ ± 8.29	65 ^a,1^ ± 5.74	76 ^a^^,1^ ± 10.40	68 ^a,1^ ± 7.48	67 ^a^^,1^ ± 6.88	90 ^a,1^ ± 8.08	93 ^a,1^ ± 9.24	91 ^a^^,1^ ± 8.47	76 ^a^^,1^ ± 9.17	77 ^a^^,1^ ± 8.38	76 ^a^^,1^ ± 8.04	*p* = 0.99
	*p* value groups	*p* = 0.99	*p* = 0.99	*p* = 0.99	*p* = 0.99	*p* = 0.99	*p* = 0.99	*p* = 0.99	*p* = 0.99	*p* = 0.99	*p* = 0.99	*p* = 0.99	*p* = 0.99	
**∑**	G_Maro_ n = 15	1.09 ^a^^,1^ ± 0.10	1.21 ^a^^,1^ ± 0.48	0.44 ^b,1^ ± 0.07	0.30 ^b,1^ ± 0.06	0.31 ^b,1^ ± 0.09	0.28 ^b,1^ ± 0.06	0.31 ^b,2^ ± 0.06	0.41 ^b,1^ ± 0.07	0.35 ^b,1^ ± 0.07	0.35 ^b,1^ ± 0.07	0.40 ^b,1^ ± 0.07	0.48 ^b,1^ ± 0.08	***p *** **= ** ***0.0006***
	G_Lido_ n = 15	1.13 ^a^^,1^ ± 0.14	1.50 ^a^^,1^ ± 0.24	0.95 ^a^^,1^ ± 0.19	0.98 ^a^^,1^ ± 0.21	0.92 ^a^^,1^ ± 0.16	0.91 ^a^^,1^ ± 0.19	0.90 ^a^^,1^ ± 0.14	0.90 ^a^^,1^ ± 0.14	0.72 ^a^^,1^ ± 0.14	0.76 ^a^^,1^ ± 0.15	0.72 ^a^^,1^ ± 0.13	0.72 ^a^^,1^ ± 0.10	*p* = 0.99
	*p* value groups	*p* = 0.99	*p* = 0.99	*p* = 0.29	*p* = 0.99	*p* = 0.34	*p* = 0.27	***p *** **= ** ***0.02***	*p* = 0.34	*p* = 0.99	*p* = 0.94	*p* = 0.87	*p* = 0.94	

1,2 = significant differences between groups, *p* ≤ 0.05. a, b, c, d, e = intragroup significant differences between assessment times, *p* ≤ 0.05. T = treatments (G_Maro_: Infusion with citrate maropitant and G_Lido_: Infusion with lidocaine). T: surgical times (T_Basal_: Basal pre-surgery; T_S_: Sedation; T_In_: Anesthetic Induction; T_Ba_: Anesthesia basal, 10 min start the anesthesia; T_P_: Pinching, clamping or placement of surgical field forceps; T_BS_: Beginning of surgery; T_RO_: ligation of the Right Ovary ligation, T_LO_: ligation of the Left Ovary ligation; T_Ut_: uterine ligation; T_Musc_: Muscle suture; T_Sut_: Suture, suturing of superficial planes and skin; T_END_: End of surgery). HR: Heart rate. T: Esophageal temperature. SAP: systolic blood pressure. MAP: mean arterial pressure. DAP: diastolic arterial pressure. **∑**: Energy.

**Table 3:** Monitoring of ventilatory parameters (RR, SpO_2_, FiO_2_, ETCO_2_), Pi, PVi, and ET_Iso_ (Mean ± SD) in the evaluation times (T) of 30 female dogs undergoing ovariohysterectomy.

Variable	Group	T_Basal_	T_S_	T_IN_	T_Surg_	*p* Value Intragroup
**RR** **(bpm)**	G_Maro_ n = 15	39 ^a^^,1^ ± 4.44	26 ^a,b,1^ ± 1.80	20 ^b,c,1^ ± 2.29	15 ^c,1^ ± 0.38	***p*** ** = ** ***0.001***
	G_Lido_ n = 15	44 ^a,1^ ± 3.97	26 ^b,1^ ± 2.15	16 ^c,1^ ± 1.65	15 ^c,1^ ± 0.37	***p *** **= ** ***0.0001***
	*p* value groups	*p* = 0.98	*p* = 0.99	*p* = 0.79	*p* = 0.99	
**SpO_2_** **(%)**	G_Maro_ n = 15	94 ^a^^,b,1^ ± 0.76	92 ^b,1^ ± 0.87	97 ^a^^,1^ ± 0.72	96 ^a^^,b,1^ ± 0.24	***p *** **= ** ***0.004***
	G_Lido_ n = 15	94 ^a^^,b,1^ ± 0.46	92 ^b,1^ ± 0.70	96 ^a^^,1^ ± 1.18	97 ^a^^,b,1^ ± 0.21	***p *** **= ** ***0.004***
	*p* value groups	*p* = 0.99	*p* = 0.99	*p* = 0.99	*p* = 0.99	
**FiO_2_**	G_Maro_ n = 15			90 ^a^^,1^ ± 1.17	92 ^a^^,1^ ± 0.23	*p* = 0.44
	G_Lido_ n = 15			90 ^a,1^ ± 0.93	92 ^a,1^ ± 0.41	*p* = 0.82
	*p* value groups			*p* = 0.99	*p* = 0.99	
**ETCO_2_** **(mmHg)**	G_Maro_ n = 15			35 ^a^^,1^ ± 1.30	37 ^a,1^ ± 0.29	*p* = 0.99
	G_Lido_ n = 15			36 ^a^^,1^ ± 1.89	37 ^a,1^ ± 0.35	*p* = 0.99
	*p* value groups			*p* = 0.99	*p* = 0.99	
**Pi** **(%)**	G_Maro_ n = 15	1.25 ^a^^,1^ ± 0.26	0.89 ^a^^,1^ ± 0.18	0.64 ^a^^,1^ ± 0.14	0.63 ^a^^,1^ ± 0.04	*p* = 0.45
	G_Lido_ n = 15	1.25 ^a^^,1^ ± 0.28	0.88 ^a,1^ ± 0.20	0.57 ^a^^,1^ ± 0.09	0.58 ^a^^,1^ ± 0.03	*p* = 0.47
	*p* value groups	*p* = 0.99	*p* = 0.99	*p* = 0.99	*p* = 0.99	
**PVi** **(%)**	G_Maro_ n = 15	32.57 ^a^^,b,1^ ± 5.66	28.93 ^a^^,2^ ± 2.29	19.07 ^a^^,b,2^ ± 1.92	16.11 ^b,2^ ± 0.36	***p *** **= ** ***0.0003***
	G_Lido_ n = 15	32.10 ^a^^,1^ ± 6.04	32.02 ^a^^,1^ ± 5.71	23.33 ^a^^,1^ ± 2.32	18.41 ^a^^,1^ ± 0.57	*p* = 0.86
	*p* value groups	*p* = 0.99	*p* = 0.99	*p* = 0.99	*p* = 0.009	
**ET_Iso_ (%)**	G_Maro_ n = 15			1.24 ^a,1^ ± 0.06	1.29 ^a,1^ ± 0.01	*p* = 0.99
	G_Lido_ n = 15			1.05 ^b,1^ ± 0.03	1.26 ^a,2^ ± 0.02	***p *** **= ** ***0.009***
	*p* value groups			*p* = 0.24	*p* = 0.94	

1, 2 = significant differences between groups, *p* ≤ 0.05. a, b, c = intragroup significant differences between assessment times, *p* ≤ 0.05. T = treatments (G_Maro_: infusion with citrate maropitant; G_Lido_: Infusion with lidocaine). T: surgical times (T_Basal_: Basal pre-surgery; T_S_: Sedation; T_IN_: Anesthetic Induction; T_Surg_: Intraoperative monitoring from the beginning of surgery until the end of the surgery. RR: Respiratory rate. SpO_2_: pulse oximetry assessed by infrared spectrophotometry. FiO_2_: Oxygen-inspired faction. ETCO_2_: end-tidal CO_2_. Pi: Perfusion index. PVi: Perfusion variability index. ET_ISO_: Total consumption of inhalation anesthetics.

## Data Availability

The original contributions presented in the study are included in the article; further inquiries can be directed to the corresponding author.
